# Quercetin Can Improve Spinal Cord Injury by Regulating the mTOR Signaling Pathway

**DOI:** 10.3389/fneur.2022.905640

**Published:** 2022-05-20

**Authors:** Xichen Wang, Yuke Fu, Benson O. A. Botchway, Yufeng Zhang, Yong Zhang, Tian Jin, Xuehong Liu

**Affiliations:** ^1^Department of Histology and Embryology, School of Medicine, Shaoxing University, Zhejiang, China; ^2^Institute of Neuroscience, Zhejiang University School of Medicine, Hangzhou, China

**Keywords:** quercetin, mTOR signaling pathway, spinal cord injury, inflammation, apoptosis, autophagy mTOR signaling pathway, autophagy

## Abstract

The pathogenesis of spinal cord injury (SCI) is complex. At present, there is no effective treatment for SCI, with most current interventions focused on improving the symptoms. Inflammation, apoptosis, autophagy, and oxidative stress caused by secondary SCI may instigate serious consequences in the event of SCI. The mammalian target of rapamycin (mTOR), as a key signaling molecule, participates in the regulation of inflammation, apoptosis, and autophagy in several processes associated with SCI. Quercetin can reduce the loss of myelin sheath, enhance the ability of antioxidant stress, and promote axonal regeneration. Moreover, quercetin is also a significant player in regulating the mTOR signaling pathway that improves pathological alterations following neuronal injury. Herein, we review the therapeutic effects of quercetin in SCI through its modulation of the mTOR signaling pathway and elaborate on how it can be a potential interventional agent for SCI.

## Introduction

Spinal cord injury (SCI) is a serious lesion of the nervous system, which may be caused by falling, shooting, and vehicle collision (1, 2). The global incidence rate of SCI is about 10.4–83 per million per year (3). SCI pathological processes are categorized into two stages: primary and secondary injuries. The primary injury often has features of bone fragments and spinal ligament tears when the spine suffers from sudden trauma that can lead to spinal fracture and vertebral dislocation (4). Initial mechanical injury can cause an imbalance of axons, blood vessels, and cell membranes at the injured site (5). Magnetic resonance imaging scans have evidenced hemorrhage, cytotoxic edema, and spinal cord swelling after the primary SCI. Moreover, the intramedullary injury is gradually expanded to the rostral and caudal sides (6). Following the primary injury, there is the instigation of the secondary injury that can lead to chemical and mechanical damages. The secondary injury causes a series of responses, such as oxidative stress, excitotoxicity, inflammation, apoptosis, autophagy, and mitochondrial dysfunction (7–11). Owing to the primary injury being an initial and irreversible process, the secondary SCI has become an important treatment time window to prevent further damage. SCI can cause neuronal atrophy and synaptic abnormalities, concomitant with inflammation, apoptosis, and autophagy. Therefore, SCI treatment should be enacted as soon as possible to control the occurrence of inflammation, reduce apoptosis, and enhance autophagy. Besides, the formation of glial scar hinders the regeneration of axons.

Quercetin is a flavonoid compound and widely exists in the roots and fruits of edible plants, such as apples and onions (12, 13). Quercetin, as a dietary supplement, is safe and has less adverse effects (14). In addition, it has features of antioxidation, anti-inflammatory, promotes autophagy, inhibits apoptosis, and improves pathological changes related to the cardiovascular, liver, and kidney (15–17). Quercetin may be a potential drug for SCI in view of the above features. Multiple studies have demonstrated that the microenvironment and cell survival after SCI can be improved by regulating a number of signaling pathways, such as the mammalian target of rapamycin (mTOR), nuclear factor-kappa B (NF-κB), mitogen-activated protein kinase (MAPK), and phosphoinositide 3-kinase (PI3K) (18–20). The mTOR signaling pathway is involved in several pathological changes, such as inflammation, autophagy, apoptosis, and oxidative stress in the wake of SCI (21, 22). Thus, regulating the mTOR signaling pathway can be an important strategy to improve SCI. The review initially expounds on the pathological changes associated with SCI and the biological roles of the mTOR signaling pathway, before exploring the relationship between SCI and the mTOR signaling pathway. Furthermore, it will endeavor to elaborate on how quercetin can regulate the mTOR signaling pathway to ameliorate SCI.

## The Pathological Changes After SCI

Signaling pathways are the significant players in inflammation, apoptosis, and autophagy after SCI. The aggravation of inflammatory response is a key factor in the development of the secondary SCI (23). Local microglia activation and leukocyte infiltration augment reactive oxygen species (ROS) to induce oxidative stress. Colony-stimulating factor 1 receptor inhibitor PLX5622 can downregulate the expressions of ROS and tumor necrosis factor-α (TNF-α) by inhibiting microglial activation and reducing the infiltration of leukocytes and monocytes (24). In addition, the products of an inflammatory response, such as interleukin-1α (IL-1α), TNF-α, and complement component 1q, can induce the activation of A1 astrocytes. The formation of A1 astrocytes is closely related to the activation of the Notch pathway. When the Notch pathway of astrocytes is activated, the Notch intracellular domain (NICD) is released into the cytoplasm, and free NICD binds to phosphorylated STAT3 to form an NICD-STAT3 complex, which then enters the nucleus and promotes the transformation of astrocytes to A1 phenotype (25). Interestingly, astrocytes can form a glial scar barrier to limit the spread of inflammation (26).

The triggering receptor expressed on myeloid cell 1 (TREM1) can be expressed in astrocytes and microglia and is overexpressed in the injured spinal cord tissue. The TREM1 activates toll-like receptors (TLRs)/myeloid differentiation primary response 88 (MyD88)/NF-κB signaling pathways to upregulate the expressions of IL-6, IL-1β, TNF-α,CCL2, and CXCL1 and intensify inflammatory response (17). The TREM1 knockout decreases the activity of TLRs/IκBα/NF-κB signaling pathway, implying that this signaling pathway activated by TREM1 can be a key player in the pathological development of SCI. Further, MyD88 activates the NF-κB kinase (IKK) inhibitor to promote the separation of the NF-κB/IκB complex and translocate p65 subunit to the nucleus. Moreover, p65 entering the nucleus can activate the release of IL-6, IL-1β, and TNF-α to aggravate inflammatory response (8). After SCI, there are the activations of a series of pathways by cell surface receptors, such as TREM1, along with the formation of ROS in cells that can instigate the inflammatory response. The advanced oxidation protein products (AOPPs) upregulate NOX4 expression to produce ROS (27). ROS can activate MAPK to promote the phosphorylation of p38 and c-Jun N-terminal kinase (JNK) and then activate NF-κB to induce the expression of NACHT-LRR-PYD domains-containing protein 3 (NLRP3), eventually causing neuroinflammatory response (28). Zinc can regulate the NLRP3 inflammatory factor by activating the nuclear factor erythroid 2-related factor 2 (Nrf2)/heme oxygenase-1 (HO-1) pathways, which in turn inhibits inflammatory reaction (29). The ROS and reactive nitrogen species (RNS) produced after SCI induce the oxidative stress response, causing destruction to protein, DNA, and lipid peroxidation (4). Thymosin beta 4 inhibits the TLR4/MyD88 signaling pathway to minimize the damage of oxidative stress induced by hydrogen peroxide to neural stem cells (30). Interestingly, the impairment of the TLRs/MyD88 signaling pathway is not only involved in mitigating inflammation, but also a significant player in antioxidant stress (30, 31). Also, the phosphorylations of PI3K and protein kinase B (Akt) activate the Nrf2/Keap1 signaling pathway to exert its antioxidant role (32). Tetramethylpyrazine can activate Akt to enhance Nrf2 expression. The activation of Nrf2 combined with the antioxidant response element (ARE), which upregulates HO-1, can mitigate inflammatory response and oxidative stress (33). Kelch-like ECH-associated protein (Keap1) can bind to Nrf2 and inhibit its activation. When Keap1 is silenced or inactivated, Nrf2 is released into the cytoplasm (34). However, p62 can destroy the ubiquitination modification of Nrf2 to separate Nrf2 from Keap1 and activate Nrf2 as well as its downstream pathways (35). The released Nrf2 then enters the nucleus and induces the expressions of HO-1, NAD(P)H-dependent quinone oxidoreductase 1 (NQO1), and glutamate-cysteine ligase catalytic subunit (GCLC) (34). Moreover, the AMP-activated protein kinase (AMPK) phosphorylation can upregulate the HO-1 expression (36). Oxidative stress induces HO-1 that can decrease the formation of ROS and RNS, and the phosphorylation of JNK, p38 MAPK, and IKK to inhibit inflammation (37).

In the wake of SCI, the damaged glial cells and neurons activate the apoptotic pathway through the mitochondria and death receptors, resulting in cell death (38). Furthermore, after SCI, lysosomal destruction causes autophagy flux disruption that increases endoplasmic reticulum stress and aggravates neuronal apoptosis (39). The ratio of beclin-1 and autophagy marker light chain 3 (LC3)II/LCI is increased after SCI (40). Interestingly, the activation of autophagy can reduce neuronal apoptosis (41). PI3K/Akt controls autophagy and apoptosis. The PI3K activated phosphorylates PIP2 and converts to PIP3, which then activates Akt (42). Also, the expressions of phosphatase and tensin homolog (PTEN) are significantly increased, which inhibits the Akt/mTOR signaling pathway and promotes apoptosis after SCI (21). PTEN inhibition can increase the activity of the downstream PI3K signaling pathway (43). Akt activates the mTOR to trigger the translation of downstream proteins by phosphorylating 4EBP1 and P70S6K (44). Berberine can inhibit the activation of mTOR/P70S6K/4EBP1, increase autophagy, and reduce apoptosis (45).

**Figure 1:**
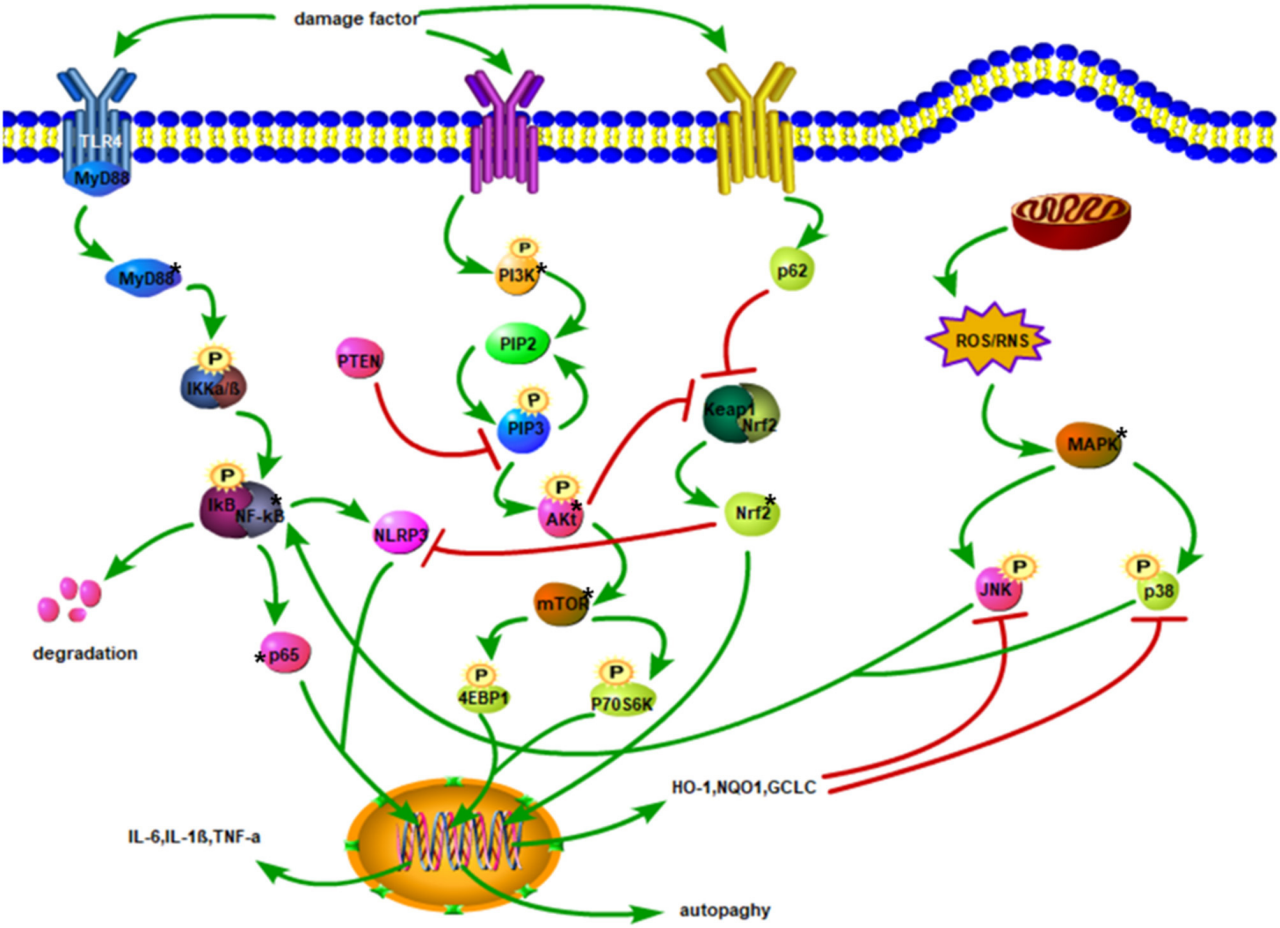
Injury factors activate TLR4/MyD88 and promote IKKα/β phosphorylation. The degradation of IκB facilitates the translocation of p65 of NF-κB to the nucleus, thereby increasing the expression of inflammatory factors. Phosphorylation of PI3K triggers the conversion of PIP2 to PIP3 and stimulates Akt phosphorylation. Akt activates mTOR to phosphorylate 4EBP1, and P70S6K to enhance autophagy. Activated p62 may inhibit Keap1 and Nrf2 complexes, promote the release of Nrf2 into the cytoplasm, and then translocate to the nucleus. After Nrf2 enters the nucleus, it upregulates the expressions of HO-1, NQO1, and GCLC, leading to oxidation impairment and hindering the phosphorylation of JNK and p38. Phosphorylated JNK and p38 activate NF-κB. Mitochondrial damage produces ROS and RNS, which activates the phosphorylation of JNK and p38 downstream of MAPK. The NF-κB stimulates NLRP3, and Nrf2 inhibits NLRP3 activation.

Understanding the pathological changes of the secondary SCI is very important for halting and treating SCI. The related mechanisms of the inflammatory response, oxidative stress, apoptosis, and autophagy are the main instigators of the secondary SCI. Inflammation, oxidative stress, and apoptosis can affect each other, and there is a close relationship between the related proteins that activate the signaling pathways of these lesions (Figure 1).

## The mTOR Signaling Pathway

### The Biological Structure of mTOR

In 1991, Heitman et al. discover the mTOR (46). Throughout evolution, the mTOR protein has been highly conserved. mTOR is a eukaryotic serine/threonine protein kinase with a 289-kDa serine, belonging to the PI3K-related kinase family (47, 48). Surprisingly, it does not have the activity of esterase kinase, but Ser/Thr protein kinase activity forms the catalytic subunits of the two different protein complexes called the mTOR complex 1 (mTORC1) and mTORC2 (49). The compositions of mTORC1 are catalytic subunits and three related proteins, Raptor, PRAS40, and mLST8/GbL. The mTORC2 includes mTOR and mLST8/GbL, along with Rictor, mSin1, and protor proteins (50).

### The Biological Function of mTOR

The mTOR signaling is paramount to the occurrence and development of several diseases, most of which are caused by mTOR overactivation. In LPS-induced depressive-like behavior, the suppression of the GluN2A-extracellular signal-regulated kinase (ERK)-mTOR signaling pathway inhibits neuroinflammation (51). Also, a significant amount of ATP produced by glycolysisactivates the mTOR signal in microglia, upregulates BDNF and TNF-α levels, and aggravates neuroinflammation (52). During the perioperative period in diabetic rats, excessive activation of the mTOR may aggravate the progression of autophagy, neuroapoptosis, and cognitive dysfunction (53). However, during development, the mTOR signal is necessary for driving the growth of myelin sheath and can promote oligodendrocytes to form myelin sheath (54). Besides, the mTOR signaling pathway plays an important role in the occurrence and repair of SCI. L-leucine promotes axonal regeneration in the SCI region by activating the mTOR signal and increases the p-mTOR expression, while also enhanced axonal crossing to the chondroitin sulfate polysaccharide region (55). Further, mTOR activation promotes neurotrophic factor-3 expression to induce the regeneration of myelinated axons (56). The abnormal activation or inhibition of the PI3K/Akt/mTOR can cause cancer, immunity, aging, metabolism, and other diseases (57–60). Considering the aforementioned studies, an in-depth investigation of the mTOR pathway can open avenues in understanding its upstream and downstream mechanisms, as well as its regulation that can be translated to improve neurological conditions.

#### The mTOR Regulates Inflammatory Response

The mTOR and its downstream (i.e., the NF-κB) factors participate in the inflammatory response. Rapamycin can decrease the occurrence of the mTOR-mediated neuroinflammation by inhibiting the overactivation of the mTOR pathway to suppress the activations of microglia and astrocytes and protect the blood–brain barrier (61). The mTOR-mediated inflammatory reaction includes two aspects: (1) The mTOR may directly induce the release of inflammatory factors and regulate the metabolism of related immune cells to instigate inflammatory response (62). Further, the mTOR signaling pathway promotes the expression of inflammatory factors, such as IL-17A, IL-17F, IL-22, and TNF-α, to aggravate inflammatory response (63, 64). (2) The activation of the related signaling pathways associated with the mTOR intensifies the progression of inflammation. For instance, amyloid-β in Alzheimer's disease can promote the phosphorylations of mTOR and NF-κB, which then triggers the occurrence of inflammation. However, metformin, the activator of AMPK, reduces amyloid-β volume and improves neuroinflammation through AMPK/mTOR/S6K/Bace1 signaling pathways (65). Meanwhile, this study also shows that the occurrence of neuroinflammation is closely related to the NF-κB activity downstream of mTOR. Besides, activating the mTOR/NF-κB signaling pathway can enhance neuroinflammation and systemic response inflammation (64). Notably, the inhibition of phosphorylated NF-κB is concomitant to the repression of the mTOR signaling pathway in the nucleus, leading to the translocation of the NF-κB to the nucleus being restricted (66). In addition, the miR-124-3p in microglial exosomes can target the PDE4B gene and inhibit the mTOR signaling pathway to alleviate neuroinflammation after brain injury (67). Therefore, the activations of mTOR and downstream NF-κB can aggravate the inflammatory response, but through cascade reactions, inhibition of mTOR activation targeted can improve inflammation.

#### mTOR Participates in Apoptosis

The mTOR signal is a key to neuronal survival, and the curtailment of the mTORC1 activity can increase neuronal apoptosis (68). As a pleiotropic cytokine, G-CSF can play a neuroprotective role in cerebral ischemia. This is due to its ability to inhibit Bax expression and CC3 activation by stimulating the mTOR/P70S6K signaling pathway and reduce the loss of pro-apoptotic neurons (69). Moreover, the Akt/mTOR is a key regulatory pathway of apoptosis, and its activation can upregulate Bcl-2 expression, reduce the ratio of Bcl-2/Bax, and minimize the apoptosis of nerve cells after repeated cerebral ischemia–reperfusion (70). The translocation of the mitochondrial p53 in the hippocampal CA1 region induces neuronal apoptosis after transient global cerebral ischemia (71). Noteworthy is that the mTOR phosphorylation negatively regulates the p53 to inhibit its phosphorylation and curtail apoptosis, which in turn can minimize the infarct volume after cerebral ischemia (72).

#### mTOR Is Involved in Autophagy

Autophagy exists in both pathological and physiological processes. Abnormal inhibition or activation of the mTOR affects autophagy activity. For instance, the inhibition of the mTOR signal can cause the limited expression of the downstream protein, P70S6K, which in turn may increase neuronal autophagy (73). Also, the inhibition of the mTOR phosphorylation promotes LC3-II expression and increases the ratio of LC3-II/LC3-I, which is concomitant with autophagy increased (74). Although neurons have moderate autophagy in the process of maintaining homeostasis, long-term overactivated autophagy can cause serious damage to neurons. The activation of the AMPK can inhibit the mTOR phosphorylation and enhance autophagy. However, alpha-lipoic acid can block the AMPK/mTOR signaling pathway to inhibit autophagy development (75). As an energy sensor of cells, the AMPK is affected by the energy changes induced by oxidative stress, which then activates autophagy. Therefore, there is also a close relationship between oxidative stress and autophagy. Besides, the AMPK can impair mTOR phosphorylation, reduce s757-ULK1 phosphorylation, promote s317-ULK1 phosphorylation, and induce autophagy in cerebral ischemia–reperfusion injury (76). Interestingly, the AMPK can negatively regulate the mTOR to promote autophagy (77). The PI3K/Akt/mTOR/P70S6K signaling pathway is also involved in the regulation of neuronal autophagy. In particular, astragalus polysaccharides inhibit the activation of the PI3K/Akt/mTOR signaling pathway, upregulate the expression of a negative regulatory protein, PTEN, and lead to the augmentation of autophagy (78). mTOR-mediated autophagy can also inhibit the activation of inflammatory factors and participate in the regulation of inflammatory response. TNF-α can activate the Akt/mTOR signaling pathway, reduce autophagy flux, and promote the transformation of microglia to M1 phenotype (79). However, the downregulation of mTOR may enhance autophagy, inhibit the activation of microglia, reduce inflammation, and promote neuronal survival (80). Although some studies have shown that the inhibition of the mTOR signal can activate excessive autophagy and cause neuronal damage, an activated autophagy through the mTOR signal pathway can also help neurons to counter the effects of inflammation and apoptosis.

## The mTOR Signaling Pathway and SCI

The regulation of the mTOR signaling pathway is important in nervous system diseases. Activating the Akt/mTOR and ERK/mTOR signaling pathways can promote the growth of neurites (81). Furthermore, G-CSF can reduce neuroinflammation and inhibit neuronal apoptosis by stimulating the mTOR/P70S6K pathway (69). Interestingly, blocking the 5-HT_6_ receptor-mediated mTOR signal activation may improve neuropathic pain and mitigate complications of cognitive impairment as evidenced in rats (82). Therefore, controlling the mTOR signaling pathway can change the processes related to inflammation, apoptosis, and autophagy in nervous system diseases.

### The Relationship Between the mTOR Signaling and Inflammation in SCI

**Figure 2:**
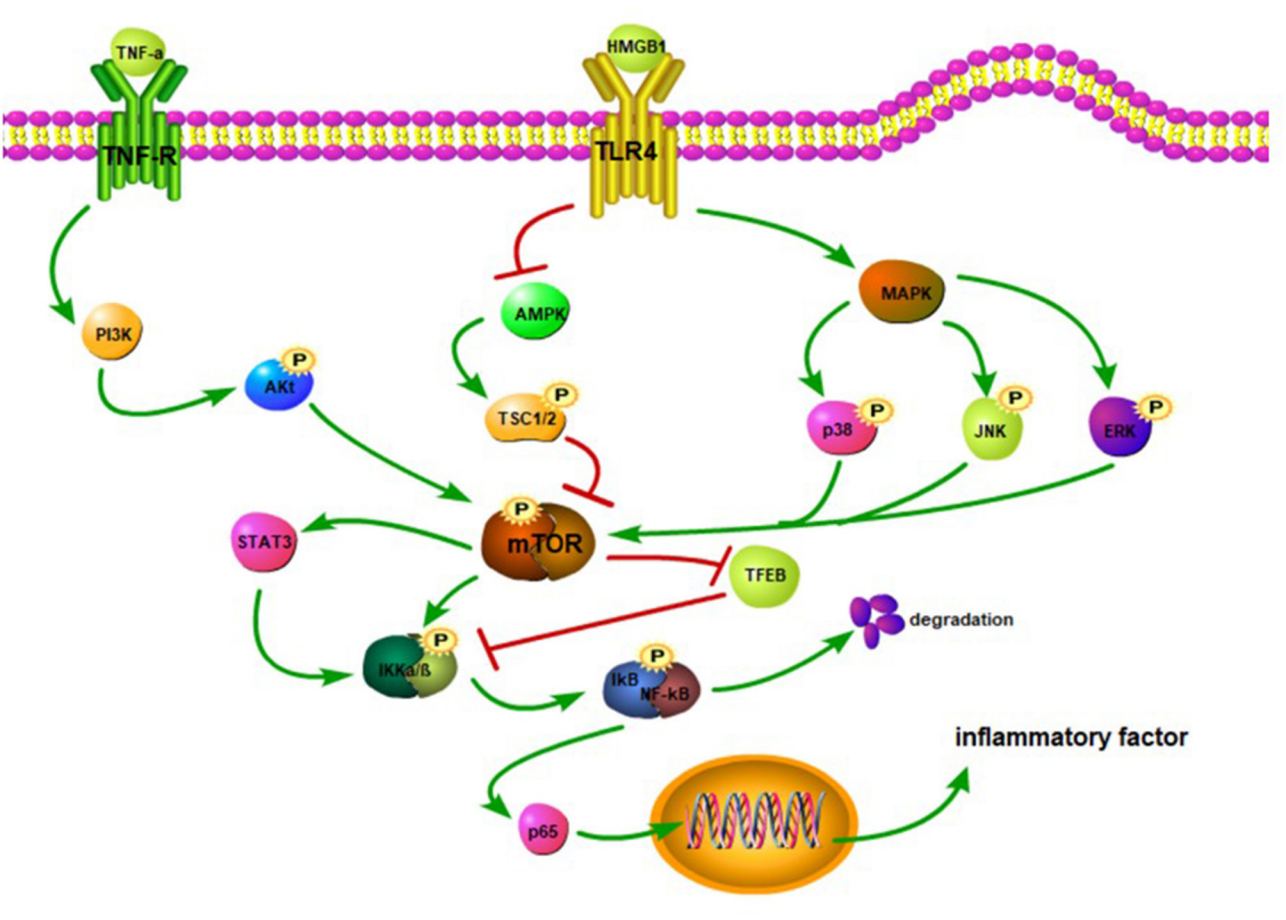
TNF-α binds to its receptor, activates PI3K, and promotes Akt phosphorylation. The Akt activates the mTOR. The HMGB1 binds to the TLR4 to inhibit the activation of the AMPK and trigger MAPK. The inhibition of the AMPK reduces the phosphorylation of TSC1/2, thereby hindering the activation of the mTOR. The MAPK promotes the phosphorylation of JNK, p38, and ERK to activate the mTOR. The mTOR stimulates STAT3 and facilitates the phosphorylation of IKKα/β. The mTOR can suppress the TFEB activation, which in turn may inhibit the IKKα/β phosphorylation. The activation of the NF-κB promotes the release of p65. When p65 is released into the cytoplasm, it translocates to the nucleus and upregulatesthe expression of inflammatory factors.

The mTOR and its downstream pathways can modulate the development of neuroinflammation after SCI (83). High-mobility group protein B1 (HMGB1) binds to the TLR4 overexpressed on the surface of activated microglia after SCI, which inhibits the AMPK phosphorylation and activates its downstream pathway, facilitating the expression of TNF-α and IL-1 to aggravate inflammatory response (84–86) (Figure 2). However, after SCI, activation of the AMPK reduces the mTOR phosphorylation level and increases the TFEB nuclear translocation level to improve the degree of injury, and this effect can be reversed by an AMPK inhibitor (87). Other studies have shown that the activation of the AMPK can phosphorylate TSC1/2 and Raptor and inhibit the mTORC1 activity (86, 88). The PI3K/Akt and MAPK signaling pathways are involved in the mTOR-mediated inflammatory response. TNF-α binds to its receptors that can activate the downstream PI3K/Akt pathway (89) (Figure 2). Activated microglia increase the NF-κB expression and IL-6 level by stimulating the PI3K/Akt/mTOR signaling pathway (90). Meanwhile, after SCI, MAPK phosphorylation promotes the phosphorylations of p38, JNK, and ERK and activates NF-κB and inflammatory response (27, 91). Therefore, the inhibition of mTOR signal can block the phosphorylation of IKKα/β and nuclear translocation of the NF-κB p65 to reduce the release of proinflammatory factors (88). Transcription factor EB (TFEB), as a downstream protein of mTOR, plays an important role in regulating inflammation. Notably, doxycycline-treated mice show significantly increased phosphorylated levels of mTOR, IKKα/β, and NF-κB, although TFEB expression is decreased (92). This suggests that mTOR may promote the nuclear translocation of NF-κB by inhibiting the TFEB (92) and thus corroborating the previous study of Wu et al. Also, mTOR can activate the STAT3 (93) and aggravate neuroinflammation and neuropathic pain after SCI (94, 95). More importantly, the STAT3 can upregulate TNF-α and IL-1 expressions through the IKK/NF-κB pathway (96).

### The Relationship Between the mTOR Signaling and Apoptosis in SCI

**Figure 3:**
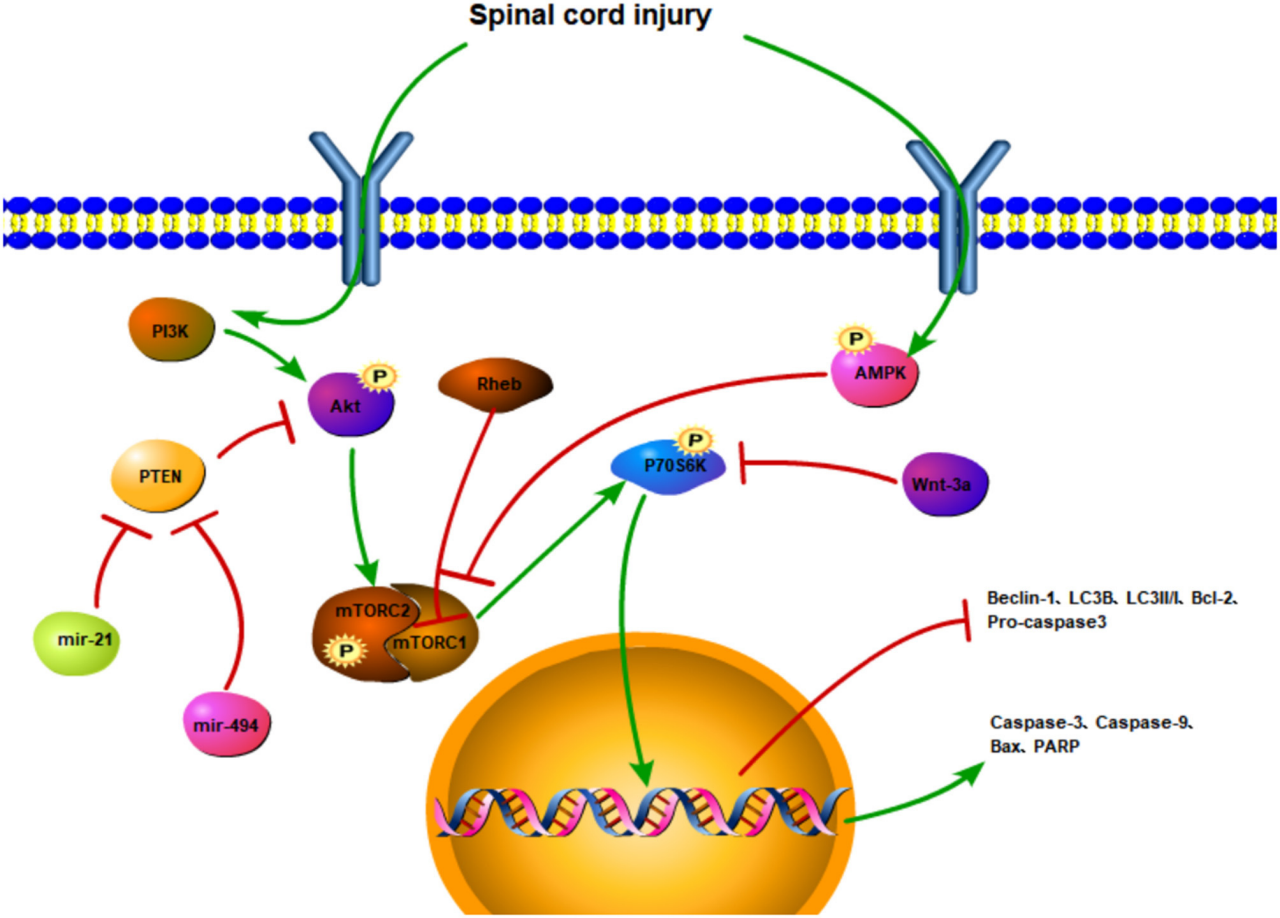
The regulatory mechanism of the mTOR signaling pathway and apoptotic autophagy after spinal cord injury. SCI activates the PI3K/Akt signaling pathway and promotes the phosphorylation of the mTOR. The PTEN inhibits the phosphorylation of the Akt and triggers the mTOR. However, miR-21 and mir-494 may hinder PTEN activation. SCI promotes AMPK phosphorylation and inhibits mTOR activation. Rheb can also inhibit mTORC1. The activation of mTOR upregulates the expressions of P70S6K, caspase-3, caspase-9, Bax, and PARP and downregulates the expressions of Beclin-1, LC3II/I, LC3B, Bcl-2, procaspase 3. Wnt-3a inhibits the phosphorylation of P70S6K.

The regulation of cell apoptosis is often accompanied by phosphorylation of the mTOR and changes in the expression of upstream and downstream factors (Figure 3). SCI affects upstream factors to instigate a downregulation in the expressions, p-Akt and p-mTOR (97). In Sprague-Dawley rats with traumatic SCI, LC3, and Beclin1 expressions are decreased along with the accumulation of autophagy substrate (p62) and ubiquitinated proteins, subsequently leading to impaired autophagy activity. Moreover, following the activation of the Rheb/mTOR signaling pathway, the expression of the pro-apoptotic protein, Bax, is downregulated, whereas the expression of the anti-apoptotic protein, Bcl-2, is upregulated, and the number of TUNEL-positive neurons is decreased (98). Similarly, autophagy is increased, with apoptosis being decreased (31). By regulating the Akt/mTOR/HIF-α signaling pathway and reducing the phosphorylation of Akt/mTOR, the apoptotic-related proteins are reduced, which in turn may inhibit the apoptosis of motor neurons.

The PTEN is a negative regulator of the Akt/mTOR pathway. By upregulating the miR-21, the overexpression of miR-21 decreases the expression of PTEN and activates the Akt/mTOR pathway. But the inhibition of the miR-21 can diminish the protective effect of neuronal apoptosis and inflammation induced by SCI. Therefore, the activation of the mir-21/PTEN/Akt/mTOR pathway may suppress neuronal apoptosis and inflammation (97). The neuroprotective effect of the miR-92a-3p is positively correlated with the activation of the PTEN/Akt/mTOR pathway after SCI (99). Also, the miR-494 can downregulate PTEN expression, improve the phosphorylations of Akt and mTOR, inhibit apoptosis, and enhance functional recovery after SCI. In addition, cleaved caspase-3, cleaved PARP, and Bax are downregulated, whereas procaspase-3, PARP, and Bcl-2 are upregulated (21).

### The Relationship Between the mTOR Signaling and Autophagy in SCI

Autophagy plays a key role in neuroprotection and nerve regeneration in SCI (Figure 3). It promotes the recruitment of autophagosomes through mTOR-independent pathways, enhances the autophagic flux of neurons, inhibits cell apoptosis through the intrinsic mitochondrial-dependent pathway, curtails the expansion of the injured cavity, mitigates neuron loss, and ultimately improves SCI (100). MicroRNA-421-3P (miR-421-3p) targets mTOR, and the miR-421-3p can bind to the 3'untranslated region (3'UTR) of the mTOR to reduce the activity of the luciferase-mtor3'UTR construct and improve neuronal autophagy (101). Increasing autophagy and reducing apoptosis can induce axonal protection after SCI (102). More so, activating the AMPK mTOR signal axis and inhibiting the mTOR stimulation may enhance the autophagy level of microglia and promote the fusion of autophagosomes and lysosomes (103). Microglia clear myelin fragments protect residual myelin and promote axonal regeneration after SCI.

Both LC3 and p62 expressions are higher after SCI. However, this is not a representation of the complete activation of autophagy flux. The lysosomal membrane protein (i.e., LAMP-2), which is closely related to autophagy degradation, is significantly decreased after SCI, implying that the downstream degradation pathway of autophagy flux is damaged after SCI. Nonetheless, autophagy flux activation through the AMPK/mTOR pathway affects the microglial polarization (104). By activating the AMPK to suppress mTOR activation and inhibit the mTOR/P70S6K signaling pathway after SCI, the levels of p-AMPK, Beclin-1, and LC3B are increased, and the expressions of p-P70S6K and p-mTOR are decreased (105), together with the significant curtailment of caspase-3, caspase-9, and Bax protein levels (106). Moreover, the upregulated expression of autophagy markers and the downregulation of apoptotic marker expressions lead to neuronal protection (107). The inhibition of mTOR signal can upregulate the TFEB expression, increase autophagy flux, activate autophagy, and exert neuroprotectiveness (108).

Wnts affect the proliferation, composition, and survival of nerve cells. The Wnt-3a can inhibit the phosphorylation level of the P70S6K, which augments Beclin-1 and LC3II/I in the neurons of spinal cord. Chondroitin sulfate proteoglycan (CSPG) increases the expression level of neurons after SCI, which has a restrictive effect on the growth and regeneration of axons, causing the inhibition of axonal regeneration of neurons. Following Wnt-3a treatment, the expression level of CSPG in the neurons of spinal cord is significantly reduced, which facilitates axonal regeneration and improves functional recovery through autophagy activation (109).

## Quercetin Regulates the mTOR Signaling Pathway to Improve SCI

Quercetin plays a neuroprotective role in neurodegeneration. In a Parkinson's disease animal model induced by rotenone, quercetin improves the degeneration of dopaminergic neurons through its anti-inflammatory and antioxidant abilities (110). Furthermore, quercetin minimizes the neurotoxicity caused by β-amyloid by activating the Sirtuin1/Nrf2/HO-1 pathway to decrease apoptosis (111). It also regulates neuronal survival and proliferation through the PI3K/Akt and ERK1/2 signaling pathways (112) and mitigates apoptosis by upregulating the expression of neurotrophic factors (NGF, BDNF) (113) to activate downstream pathways (114). Interestingly, quercetin can promote the expression of LC3II by inhibiting mTOR phosphorylation to enhance autophagy activity and promote the recovery of motor function (115). It can also activate the mTOR pathway to improve cognitive impairment after sleep deprivation (116). However, in severely injured neurons, low-dose quercetin may promote oxidation (112). Although a study shows quercetin to promote oxidative stress, multiple studies have demonstrated that it can improve nerve injury, especially through the mTOR signaling pathway.

### The Biological Roles of Quercetin

The therapeutic effect of quercetin has been widely studied in different fields, such as cancer, cardiovascular, and kidney (15, 16, 117). Quercetin promotes the nuclear translocation of transcription factor EB, activates lysosomes, causes the accumulation of iron, and leads to the death of cancer cells (115). Quercetin can activate the FXR1/TGR5 signaling pathway to improve lipid metabolism in non-alcoholic fatty liver disease (118). In addition, quercetin may inhibit the NADPH oxidase by upregulating the HO-1 expression and minimizing ROS formation (119) to protect the kidney and pancreas (120). Besides, quercetin is significantly involved in the treatment of neurodegenerative diseases (Table 1). Quercetin increases the level of Nrf2 mRNA expression and promotes AMPKα and SIRT1 expressions to reduce the generation of ROS (133). Also, quercetin increases ERK and Akt phosphorylation by inhibiting protein threonine and serine–threonine phosphatase activity, thereby reducing neuronal apoptosis after cerebral ischemia–reperfusion injury (134). Mitochondrial fragmentation can lead to neuronal degeneration. However, quercetin can upregulate the expression of H2AX and mitochondrial transcription factor A to minimize the damages in the mitochondrial structure and activity, while also reducing axonal and neuronal detriments (135). Also, quercetin can reduce the damage of myelin loss to white matter after cerebral hypoperfusion by promoting the secretion of IL-10 by M2-like microglia (136). Quercetin decreases the apoptosis of cortical neurons, improves degenerative lesions of the central nervous system, and plays a neuroprotective role in Alzheimer's disease (137). Quercetin also improves the gray and white matter injuries after SCI, which may indicate its potentiality for SCI treatment. Furthermore, quercetin improves neuroinflammation by reducing the activation of STAT1 and NF-κB pathways to impair the polarization of macrophages or microglia to M1 (138) and mitigates the activation of astrocytes and microglia (135). After SCI, the death of oligodendrocytes causes demyelination, resulting in permanent neurological dysfunction. However, the use of quercetin mitigates the necrosis of oligodendrocytes by improving the living environment of oligodendrocytes, while also reducing myelin loss and axonal damage (138). The hemorrhage causes hemoglobin decomposition to form Fe^2+^, which then induces apoptosis and oxidative stress in the area of SCI. Nonetheless, quercetin can chelate Fe^2+^ and remove free iron to improve acute injury after SCI (139). Quercetin improves the electrophysiological function after acute SCI, increases the 5-HT-positive nerve fibers, and promotes axonal regeneration (140). Besides, in the early stage after acute SCI, the reactive astrocytes activated by quercetin stabilize the central nervous system and play a positive role in SCI. Also, isoquercetin, an analog of quercetin, inhibits the mitochondrial and endoplasmic reticulum stress in cells, reduces neuronal apoptosis, and exerts neuroprotection in acute SCI (141).

**Table 1:** Therapeutic effect of quercetin on neurodegenerative diseases.

**Disease**	**Species**	**Mode of administration**	**Results**	**References**
Alzheimer's disease	Rat	Intraperitoneal injection	Quercetin can improve spatial memory impairment and reduce oxidative stress in rats.	(121)
Alzheimer's disease	Mouse	Oral adiministration	Quercetin reduced β-amyloidosis in amygdala, improved learning ability and delayed cognitive impairment.	(122)
Alzheimer's disease	Rat	Gastric tube administration	Quercetin can reduce astrocyte proliferation and reduce the formation of the intracellular neurofibrillary tangles and amyloid plaques.	(123)
Parkinson's disease	Mouse	Intragastric administration	Quercetin can increase the expression of brain-derived neurotrophic factor and increase the biogenesis of mitochondria in dopaminergic neurons.	(124)
Parkinson's disease	Rat	Intragastric administration	Quercetin reduces neuronal loss and activates mitochondrial autophagy to reduce mitochondrial damage.	(125)
Parkinson's disease	Rat	Intraperitoneal injection	Quercetin can reduce endoplasmic reticulum stress and increase autophagy, thereby can inhibit neuronal apoptosis.	(126)
Amyotrophic lateral sclerosis	Rat	Intragastric administration	Quercetin can inhibit oxidative stress and up regulate Bcl-2 to inhibit apoptosis.	(127)
Brain injury	Rat	Intragastric administration	Quercetin can inhibit apoptosis, reduce the proliferation of microglia and astrocytes to alleviate inflammatory response.	(128)
Brain injury	Mouse	Intraperitoneal injection	Quercetin reduces mitochondrial oxidative stress in damaged brain regions, increases mitochondrial biological activity and protects neurons.	(129)
Cerebral ischemia	Rat	Intraperitoneal injection	Quercetin reduced lipid peroxidation in cerebral cortex and Cu/Zn ratio in injured area.	(130)
Cerebral ischemia	Rat	Intraperitoneal injection	Quercetin increases the expression of thioredoxin, which reduces neuronal apoptosis and oxidative stress.	(131)
Huntington's disease	Rat	Intraperitoneal injection	Quercetin inhibits the proliferation of microglia in the lesion area and improves motor coordination disorder.	(132)

### Quercetin Can Alleviate Inflammatory Response by Regulating the mTOR Signaling in SCI

**Figure 4:**
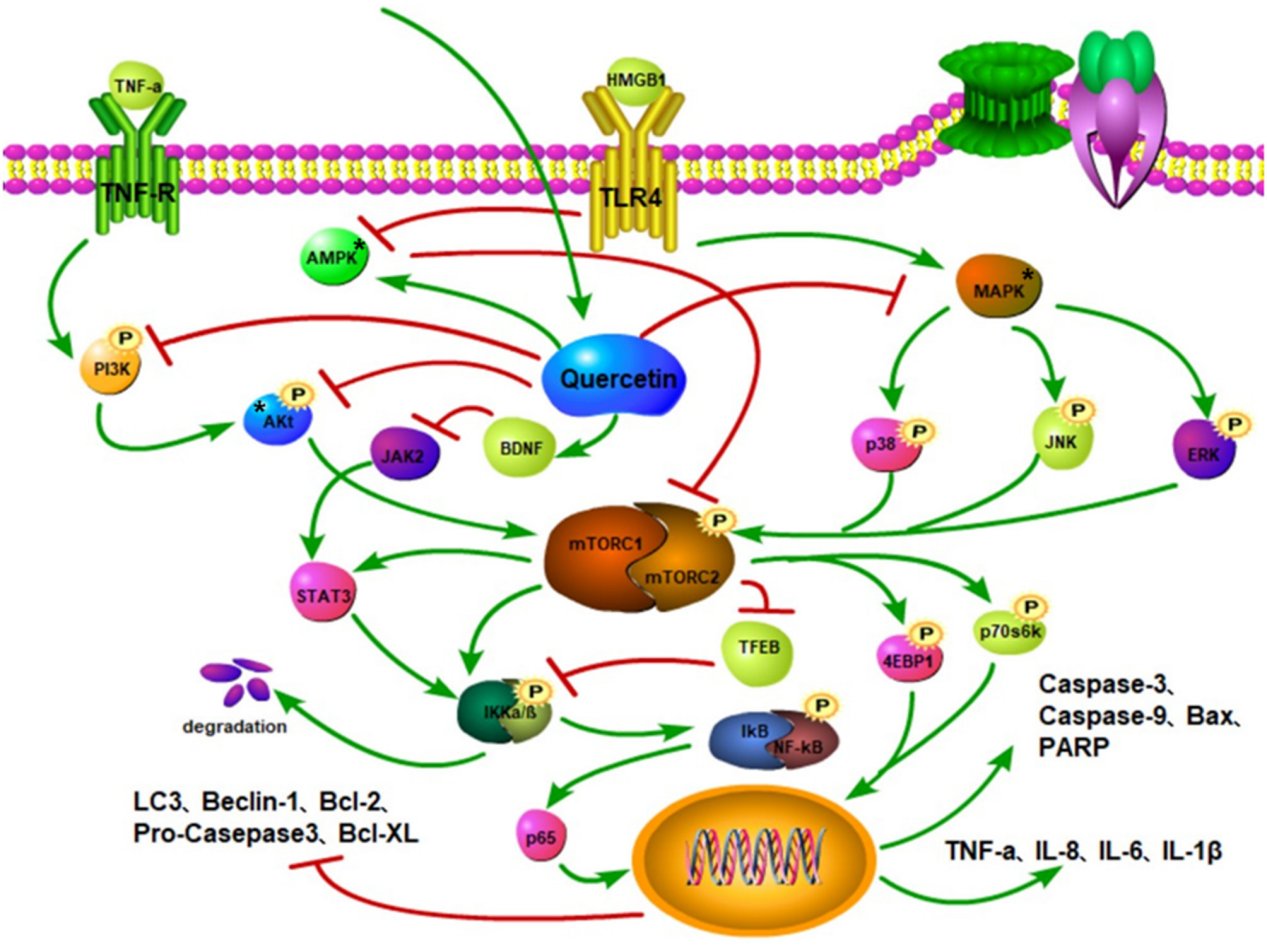
Quercetin inhibits the phosphorylation of PI3K and Akt and mitigates the activation of mTOR. Quercetin activates the AMPK to inhibit mTOR activation and stimulate autophagy. Quercetin suppresses the MAPK activation to curtail the phosphorylations of p38, JNK, and ERK, thereby reducing mTOR activation, while also increasing the ratio of LC3-II and autophagosomes. It also restricts BDNF to inhibit JAK activity and STAT3 activation. The inhibition of the mTOR activity reduces the activations of STAT3 and IKKα/β to promote the TFEB expression. The phosphorylations of IκB and NF-κB complexes attenuate the nuclear translocation of p65 and downregulate the expressions of pro-inflammatory factors, such as TNF-α, IL-8, and IL-6. Quercetin downregulates Bax, caspase-9, and caspase-3 protein expressions, inhibits the phosphorylations of Akt, mTOR, P70S6K, and 4E-BP1, and increases Beclin1, Bcl-2, and LC3-II protein levels through the PI3K/Akt/mTOR signaling pathway.

Quercetin is involved in the regulation of mTOR-mediated inflammatory response and therefore can be a potential drug that improves pathological changes after SCI. For instance, TNF-α upregulates the expression of NF-κB and AP-1 to aggravate inflammation. However, quercetin treatment reverses this effect (142). Quercetin can inhibit the activation of MAPK, reduce the phosphorylation of JNK, p38, and ERK to inhibit the mTOR phosphorylation, leading to the downregulated expressions of IL-6, IL-1β, and TNF-α that alleviates inflammatory reaction (143, 144). Moreover, quercetin downregulates the TNF-α expression, inhibits the activation of hypothalamic–pituitary–adrenocortical axis, reduces neuroinflammation after stress, and improves cognitive impairment in mice (145). Following SCI, quercetin minimizes the expression of NLRP3 inflammation-related protein (146) and upregulates the BDNF expression to block the JAK2/STAT3 signaling pathway (140) (Figure 4). Noteworthy is that the mTOR/STAT3 pathway is involved in LPS-induced inflammatory response (147). Hence, quercetin can reduce inflammatory response by regulating the phosphorylation of mTOR and STAT3.

Quercetin can also inhibit the Akt/mTOR signaling pathway to decrease the mTOR phosphorylation (148), along with curtailing the phosphorylation of PI3K and Akt by inhibiting the formation of the TLR4/MyD88/PI3K complex, subsequently reducing the nuclear translocation of NF-κB (149) (Figure 4). Far more, the anti-inflammatory effect of quercetin is demonstrated to be time- and dose-dependent (149). Interestingly, quercetin can activate the AMPK pathway to inhibit the mTOR and reduce nuclear translocation of the NF-κB to improve inflammation (144, 150, 151).

### Quercetin Can Inhibit Apoptosis and Upregulate Autophagy Reaction by Regulating the mTOR Signaling in SCI

Apoptosis and autophagy are the key players in SCI repair. Quercetin promotes the motor and electrophysiological functional recovery, astrocytic activation, and axonal regeneration after acute SCI (140). Moreover, it minimizes the formation of cavities at the injured site and pathological damage to nerve tissue, improves post-traumatic spinal cord edema (152), increases neuronal survival, promotes nerve regeneration (153), preserves the injured spinal cord tissue (154), and facilitates motor function recovery (139). The level of S-100β in serum is associated with the degree of nerve injury, neuroprotection, and regeneration. Quercetin significantly increases the level of S-100β that improves nerve injury (155). Following SCI, the employment of quercetin alters apoptotic and autophagy protein factors through the impairment of the mTOR signaling pathway (Figure 4). Also, quercetin considerably inhibits the augmentation of phosphorylated p38MAPK (p-p38MAPK) (156) and decreases the levels of TNF-α, IL-1β, Bcl-2, Bax, caspase-3, and caspase-9 expressions (157), leading to reduce spinal cord inflammation and impair apoptosis. The inhibition of the p38MAPK activity results in the mTOR diminution and causes an increase in autophagic activity (158). Both P70S6K and 4E-BP1, which are located in the downstream of mTOR, are dephosphorylated and increased caspase-3, caspase-9, and cleaved PARP (159). In nerve tissues, quercetin hinders the kinase activity of the mTOR in a dose-dependent manner, which improves the function of related signal axes and enhances cell degradation and self-renewal capabilities (160). Besides, quercetin mitigates the phosphorylated level of Akt, mTOR, and P70S6K and promotes motor function recovery, axonal regeneration, and energy metabolism following SCI. Also, it can inhibit apoptosis by activating the PI3K/Akt signaling pathway to increase Bcl-2 protein expression and decrease Bax protein expression (161). In addition, quercetin activates autophagy through the AMPK signaling pathway (162).

## Conclusion and Future Prospect

The mTOR is involved in the pathological processes associated with SCI, and the regulation of the mTOR can significantly improve inflammation, apoptosis, and autophagy after SCI. Quercetin can play a neuroprotective role in nervous system diseases. Subsequent to SCI, quercetin can minimize inflammation and apoptosis and enhance autophagy by modulating the mTOR signaling pathway. This can reduce the secretion of inflammatory factors, inhibit microglial activation, increase autophagy processes, and lessen cell damage. Based on the aforementioned therapeutic effects, quercetin may be an interventional agent that regulates the mTOR signaling pathway in the treatment of SCI. Nevertheless, the effect and specific mechanism of quercetin in different stages of SCI still need further investigation using different animal models before it is employed in the clinical setting.

## Author Contributions

XL designed the study. XW, YF, BB, YuZ, YoZ, TJ, and XL prepared the first draft of the manuscript and revised the manuscript. All authors approved the final version of the manuscript.

## Funding

This study was supported by the Natural Science Foundation of Zhejiang Province (No. LY19H170001).

## Conflict of Interest

The authors declare that the research was conducted in the absence of any commercial or financial relationships that could be construed as a potential conflict of interest.

## Publisher's Note

All claims expressed in this article are solely those of the authors and do not necessarily represent those of their affiliated organizations, or those of the publisher, the editors and the reviewers. Any product that may be evaluated in this article, or claim that may be made by its manufacturer, is not guaranteed or endorsed by the publisher.

## Abbreviations

SCI: spinal cord injury
mTOR: the mammalian target of rapamycin
NF-κB: nuclear factor-kappa B
MAPK: mitogen-activated protein kinase
PI3K: Phosphoinositide 3-kinase
ROS: reactive oxygen species
TNF-α: tumor necrosis factor alpha
IL-1α: interleukin-1α
NICD: the Notch intracellular domain
TREM1: triggering receptor expressed on myeloid cells 1
TLRs: toll-like receptors
MyD88: myeloid differentiation primary response 88
IKK: inhibitor of nuclear factor kappa-B kinase
IκB: inhibitor of NF-κB
AOPPs: advanced oxidation protein products
JNK: c-Jun N-terminal kinase
NLRP3: NACHT-LRR-PYD domains-containing protein 3
Nrf2: nuclear factor erythroid 2-related factor 2
HO-1: Heme oxygenase-1
ARE: antioxidant response element
Keap1: the Kelch-like ECH-associated protein
NQO1: NAD(P)H-dependent quinone oxidoreductase 1
GCLC: glutamate-cysteine ligase catalytic subunit
Akt: protein kinase B
AMPK: AMP-activated protein kinase
PTEN: phosphatase and tensin homolog
mTORC1: mTOR complex 1
RA: rheumatoid arthritis
TFEB: Transcription factor EB
LC3: light chain 3
CSPG: Chondroitin sulfate proteoglycan
ERK: extracellular signal-regulated kinase.
